# 

**Published:** 1870-09

**Authors:** 


### Books and Pamphlets Received.

The Practice of Medicine. By Thomas Hawkins Tanner, M. D., F.R.S. Fifth American, from the sixth London edition, enlarged and thoroughly revised.—Philadelphia, Lindsay & Blakiston, 1870. Received through and for sale by Breed, Lent & Co.

Constitution and By-Laws of the Medical Society of the district of Columbia, with the Act of Incorporation and List of Members.—Washington: W. H. Moore, 511 Eleventh Street.

A Guide to the Examination of the Urine.—By Dr. Wickham Legg.

